# Bundling up DNA

**DOI:** 10.7554/eLife.37234

**Published:** 2018-05-17

**Authors:** Susan A Gerbi

**Affiliations:** Department of Molecular Biology, Cell Biology and BiochemistryBrown UniversityProvidenceUnited States

**Keywords:** satellite DNA, chromocenter, micronuclei, pericentromeric heterochromatin, mitosis, D1/HMAG1, *D. melanogaster*, Mouse

## Abstract

Structures known as chromocenters, comprising satellite DNA and proteins such as D1 or HMGA1, help to contain DNA inside the nucleus between cell divisions.

**Related research article** Jagannathan M, Cummings R, Yamashita YM. 2018. A conserved function for pericentromeric satellite DNA. *eLife* **7**:e34122. doi: 10.7554/eLife.34122

A distinctive key feature of all eukaryotes – a large group of organisms that includes fungi, plants and animals – is that their genetic material is packaged within the nucleus, a cellular compartment delimited by a double membrane. This ‘nuclear envelope’ physically separates transcription and translation, the two genetic steps needed to make new proteins (Dahlberg and Lund, 2012; Pederson, 2013).

When cells divide, the chromosomes inside the nucleus condense and the nuclear envelope breaks down so that the genetic material can move to the daughter cells. When cell division is complete, the nuclear envelope forms again. During the ensuing interphase (the period between two cell divisions), the chromosomes decondense, the genome can be duplicated and the genes expressed. However, a single human cell contains up to two meters of DNA: is the nuclear envelope on its own sufficient to contain the genome inside the nucleus during interphase? Now, in eLife, Yukiko Yamashita and her group at the University of Michigan – including Madhav Jagannathan as first author – report how structures known as chromocenters help to keep the genome within its nuclear casing between cell divisions (Jagannathan et al., 2018).

Found in a wide range of organisms, chromocenters are masses of heterochromatin – densely packed DNA and proteins – that come together during interphase (Figure 1A; Jones, 1970; Fransz et al., 2002). Yet, despite their widespread occurrence, the role of the chromocenters remains enigmatic. Here, Jagannathan et al. explore their function by studying a group of molecules called multi-AT-hook proteins, with a focus on the protein D1 in fruit flies and HMGA1 in mice.

**Figure 1.**
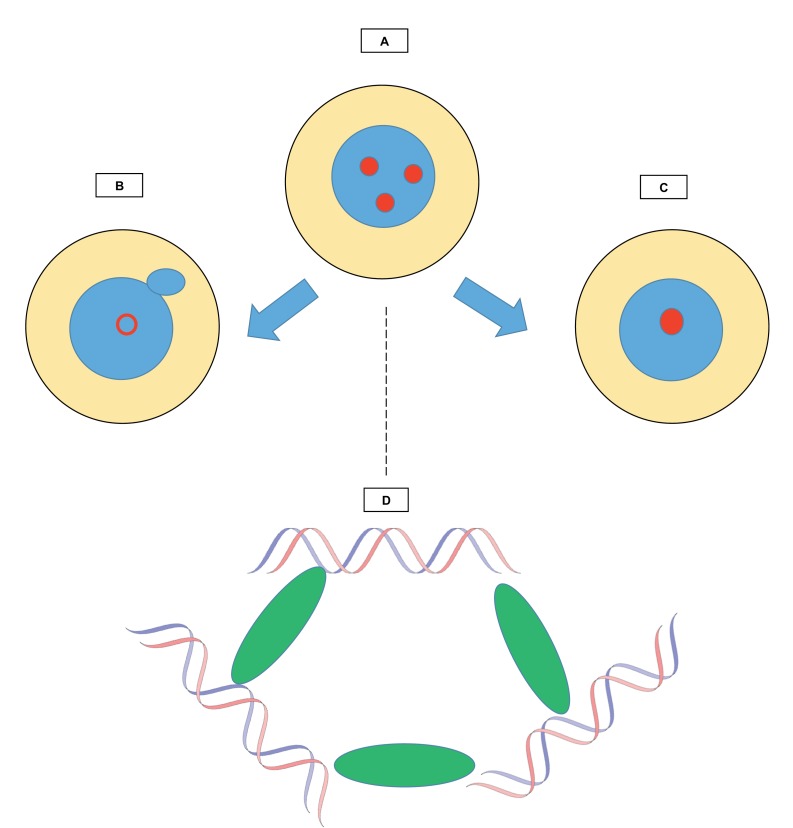
Relationships between chromocenters and multi-AT-hook proteins. The figure shows how structures known as chromocenters (red circles) form in the nucleus (blue) of a cell (yellow). (**A**) During interphase, the period between two cell divisions, certain regions called satellite DNA come together in the nucleus to form chromocenters. The work by Jagannathan et al. explores the role of multi-AT-hook proteins in the creation of these structures. (**B**) When multi-AT-hook proteins are depleted from the cell, the chromocenters are disrupted (hollow red circle), and structures (little blue circle) bud off from the nuclei, forming small independent ‘micronuclei’ that contain portions of the genome. (**C**) When multi-AT-hook proteins are overexpressed, the chromocenters coalesce. (**D**) Magnified image of one chromocenter: multi-AT-hook proteins (green ovals) bundle up satellite DNA (blue and red strands of the DNA double helix) from three different interphase chromosomes.

Chromocenters contain ‘pericentromeric regions’ of DNA, comprising highly repetitive, non-coding ‘satellite’ DNA sequences (Botchan et al., 1971; Gall et al., 1971; Peacock et al., 1974; Guenatri et al., 2004). These sequences evolve rapidly, and without any apparent selection. The multi-AT-hook proteins can bind to pericentromeric satellite DNA, and these proteins are present in chromocenters during interphase. Jagannathan et al. conducted experiments in fruit flies and in mouse cells, and showed that when these proteins were absent, the chromocenters were disrupted. Removing D1 and HMGA1 also led to the formation of micronuclei, small structures composed of DNA enclosed in nuclear membranes (Figure 1B).

One possibility is that micronuclei appeared because chromosomes had lagged during cell division and were not included in the nuclei. However, Jagannathan et al. showed that, rather than being due to lagging chromosomes, micronuclei formed during interphase and budded off from nuclei in a process known as blebbing. Indeed, when micronuclei were present, the cells showed defects in their nuclear envelope and holes in their nuclear lamina, a network of fibers that lines the inside of the membrane of the nucleus. In turn, micronuclei formation can lead to DNA breakage and even cell death.

When the fruit fly D1 protein was artificially overexpressed in mouse cells, fewer chromocenters were observed. This suggests that more clustering had occurred (Figure 1C), and also demonstrated that D1 could bind to pericentromeric regions in mice. This is surprising because D1 and HMGA1 attach to different DNA sequences. However, the DNA sequences recognized by D1 and HMGA1 are both AT-rich and hence can both bind to AT-hook proteins. Moreover, when D1 was artificially tethered to DNA at sites it does not normally bind to, these regions were brought to the chromocenters. Jagannathan et al. concluded that in both mice and fruit flies, multi-AT-hook proteins attach to satellite DNA on different chromosomes, thereby bundling the DNA sequences together and bringing them to the chromocenters (Figure 1D).

Using high-resolution microscopy, Jagannathan et al. also observed chromatin fibers that contain satellite DNA and the proteins D1 (in fruit flies) or HMGA1 (in mice). These fibers connected different chromosomes. This suggests that during interphase, the chromocenters keep the genome within the nucleus by gathering pericentromeric DNA from different chromosomes. Evolution would select for satellite DNA that binds a bundling protein, but not for the satellite sequence itself, which can rapidly diverge between species (Jagannathan and Yamashita, 2018; Jagannathan et al., 2018).

The study by Jagannathan et al. is a starting point to explore the formation of micronuclei and the loss of some genomic DNA. In that regard, investigating the similarities between micronuclei and structures known as karyomeres could be enlightening. Karyomeres are single or groups of a few chromosomes that become enclosed by the nuclear envelope at the end of mitosis. Subsequently, the karyomeres fuse and form a single, large nucleus. An intriguing hypothesis would be that micronuclei form by reversing the pathway of karyomere fusion.

Blebbing leads to the formation of micronuclei, but the details of this process are still unclear. When does blebbing take place during interphase – before, during or after DNA replication, or at any point before cell division? Are the observed defects of the nuclear membrane the cause or the result of formation of micronuclei? Also, little is known about the genetic material inside the micronuclei, such as whether it consists of entire chromosomes or only fragments, and whether certain DNA sequences are more likely to be present.

There are a few cases where chromosomes are discarded as a normal part of development. For example, both male and female fungus gnats dispose of one paternal X chromosome during interphase of embryonic germ cells. These insects also remove all their ‘germ-line limited chromosomes’, chromosomes which only exist in the reproductive cell lineage (Berry, 1939; Berry, 1941; Rieffel and Crouse, 1966). Perhaps micronuclei could be a way for these organisms to perform such key genetic processes. The work reported by Jagannathan et al. establishes the foundation on which to address these fascinating biological questions.
